# A discriminant analysis prediction model of non-syndromic cleft lip with or without cleft palate based on risk factors

**DOI:** 10.1186/s12884-016-1116-4

**Published:** 2016-11-23

**Authors:** Huixia Li, Miyang Luo, Jiayou Luo, Jianfei Zheng, Rong Zeng, Qiyun Du, Junqun Fang, Na Ouyang

**Affiliations:** 1 Department of Maternal and Children Health, Xiangya School of Public Health, Central South University, No.110, Xiangya Road, Kaifu District, Changsha, 410008 Hunan Province China; 2 Department of Child Health Care, Hunan Provincial Maternal and Child Health Care Hospital, No.53, Xiangchun Road, Kaifu District, Changsha, 410008 Hunan Province China; 3 Department of Epidemiology, Saw Swee Hock School of Public Health, National University of Singapore, 21 Lower Kent Ridge Road, Singapore, 119077 Singapore; 4 Department of Emergency and Intensive Care Medicine, Second Xiangya Hospital, Central South University, No.139, Central Renminzhong Road, Furong District, Changsha, 410011 Hunan Province China; 5 Department of Pharmacy, Xiangya Hospital, Central South University, No.87, Xiangya Road, Kaifu District, Changsha, 410008 Hunan Province China; 6 Department of Health Care, Hunan Provincial Maternal and Child Health Care Hospital, No.53, Xiangchun Road, Kaifu District, Changsha, 410008 Hunan Province China; 7 Department of Hospital Infection-Control, Second Xiangya Hospital, Central South University, No.139, Central Renmin Road, Furong District, Changsha, 410011 Hunan Province China

**Keywords:** Non-syndromic cleft lip with or without cleft palate, Prediction model, Discriminant analysis, Risk factors

## Abstract

**Background:**

A risk prediction model of non-syndromic cleft lip with or without cleft palate (NSCL/P) was established by a discriminant analysis to predict the individual risk of NSCL/P in pregnant women.

**Methods:**

A hospital-based case–control study was conducted with 113 cases of NSCL/P and 226 controls without NSCL/P. The cases and the controls were obtained from 52 birth defects’ surveillance hospitals in Hunan Province, China. A questionnaire was administered in person to collect the variables relevant to NSCL/P by face to face interviews. Logistic regression models were used to analyze the influencing factors of NSCL/P, and a stepwise Fisher discriminant analysis was subsequently used to construct the prediction model.

**Results:**

In the univariate analysis, 13 influencing factors were related to NSCL/P, of which the following 8 influencing factors as predictors determined the discriminant prediction model: family income, maternal occupational hazards exposure, premarital medical examination, housing renovation, milk/soymilk intake in the first trimester of pregnancy, paternal occupational hazards exposure, paternal strong tea drinking, and family history of NSCL/P. The model had statistical significance (lambda = 0.772, chi-square = 86.044, df = 8, *P* < 0.001). Self-verification showed that 83.8 % of the participants were correctly predicted to be NSCL/P cases or controls with a sensitivity of 74.3 % and a specificity of 88.5 %. The area under the receiver operating characteristic curve (AUC) was 0.846.

**Conclusions:**

The prediction model that was established using the risk factors of NSCL/P can be useful for predicting the risk of NSCL/P. Further research is needed to improve the model, and confirm the validity and reliability of the model.

## Background

Non-syndromic cleft lip with or without cleft palate (NSCL/P) is the most common craniofacial congenital anomaly. The incidence of the anomaly worldwide is 0.3 to 1.9 per thousand live births [1–3], and the average incidence is 0.8 per thousand live births [1]. China is one of the countries with a high incidence of NSCL/P, at 1.3 per thousand live births [4], which is higher than the world’s average level. The anomaly not only causes facial deformity in children, but it also influences their sucking, swallowing, and the development of language and hearing, and even results in psychological problems [5–7]. It increases the mental and financial burden on the subjects and their families [8], having a direct impact on their quality of life [9]. Thus, the prevention of NSCL/P is now regarded as an important public health issue in world.

Due to the complicated pathogenesis of the disease, the etiology of NSCL/P has not been fully understood, and the existing evidence today suggests a multifactorial inheritance for this anomaly, with both genetic and environmental causal factors. Recently, most studies have focused on the identification of risk factors of NSCL/P. Many epidemiological studies have confirmed that maternal age [10–12], maternal educational level [2, 13], family income [13, 14], abnormal reproductive histories [15], family history [14–16], history of infection during pregnancy [17], medication use during pregnancy [18, 19], ambient environment pollution [20], parental occupational hazards exposure [21–23], maternal nutrient intake [23–26], and maternal lifestyle factors (alcohol drinking, smoking) [27–29] are associated with NSCL/P. However, an individual risk prediction tool for NSCL/P has not been reported. Predicting an individual’s risk based on a range of presumed risk factors is fundamental to prevent NSCL/P, which can provide ancillary information for prenatal diagnosis of NSCL/P.

Previous studies have shown that a statistical prediction model based on the risk factors is an effective method for predicting the individual risk of disease, such as coronary heart disease, hypertension, and type-2 diabetes mellitus (DM) [30–32]. For example, Qian et al. [32] develop a prediction model of type-2 DM using an artificial neural network model with a sensitivity of 93.3 % and a specificity of 61.1 %, suggesting that the model can accurately predict the risk of type-2 DM.

However, there is rare research about individual risk prediction of birth defects. In our previous study, we used a decision tree to predict the risks of total birth defects and congenital heart disease based on risk factors in the first trimester of pregnancy [33, 34]. The predictors of the two models include maternal sociodemographic characteristics, family histories of birth defects, environmental risk factors, and nutrition for pregnancy. The accuracy rates of the two prediction models are 83.7 and 82.8 %, respectively. Birth defects risk prediction is a field worthy of study, and should be expanded to other types of birth defects. At present, there is no report about NSCL/P risk prediction. To predict the risk of NSCL/P in pregnant women, here we construct an NSCL/P risk prediction model by discriminant analysis based on risk factors.

## Methods

### Subjects

We conducted a hospital-based case–control study on mothers whose fetuses or neonates were between the 28th week of gestation and the 7th day after birth (including live births, fetal deaths, and stillbirths) and were diagnosed with non-syndromic cleft lip with or without cleft palate (NSCL/P) between July 2012 and June 2013 in 52 birth defects’ surveillance hospitals in Hunan Province, China. Mothers who delivered normal infants at the same hospitals as the cases were randomly selected as the controls. Additionally, the interval of the birth dates between the normal infants and the patients with NSCL/P was no more than 1 month. Those mothers were aged 20–45 years. The diagnosis of NSCL/P was performed by the clinical geneticists of those birth defects surveillance hospitals. Infants with chromosomal anomalies and other birth defects of known aetiology were excluded from the survey. Infants with cleft palate only were also excluded from the study. Those who could not cooperate with the survey were excluded from the study.

In this hospital-based study, the control-to-case ratio was 2:1, due to the relatively small number of cases and a large number of potential controls to be selected from the birth defects’ surveillance hospitals. In case of few cases, using the control-to-case ratio of 2:1 could ensure the necessary statistical power to identify important predictors.

### Data collection

The survey was conducted by obstetricians and gynecologists who were also trained investigators using the unified questionnaire with the participants in person by face to face interview. The unified questionnaire was designed by the experts on our research team, and was modified based on the pilot study. The contents of questionnaire were classified 5 categories and 28 variables, including sociodemographic characteristics of the mothers, economic status of their families, family histories, conditions of the mothers from 6 months before conception through the first trimester of pregnancy and characteristics and conditions of the fathers.

#### Measurements of variables

*Sociodemographic characteristics and family income* Maternal age was classified into four scales (years): 20–24, 25–29, 30–34, ≥35. Maternal education level was classified into three categories: primary school and below, middle school, college and above. Maternal occupations included farmers, migrant workers, employers/managers, workers, staffs in administrative institutions, and housewives or else. Family income was classified into four scales (yuan/year/person): ≤5000, 5001–10,000, 10,001–15,000, >15,000.

*Family histories* Family histories of NSCL/P were defined as one or more first relatives of one person suffering from NSCL/P. In this study, family histories of NSCL/P were included the family histories of mother and father. Abnormal reproductive histories referred to the histories of stillbirth, spontaneous abortion, or birth defect.

*Conditions of the mothers* In this study, most variables were dichotomies, collected from the questionnaire using the questions with answers yes or no, including occupational hazards exposure, premarital medical examination, chronic disease, upper respiratory tract infection, reproductive system infection, complications of pregnancy, contraceptive intake, folic acid intake, housing renovation and strong tea drinking. The exposure time of maternal variables was defined as from 6 months before conception through the first trimester of pregnancy. Occupational hazards exposure was defined as having been exposed to those toxic and hazardous substances in their workplace, including organic solvents (benzene, toluene, n-hexane, methyl alcohol, glycol ether), noxious gases (hydrogen sulfide, ammonia, formaldehyde, sulfur dioxide, ozone), heavy metals (Pb, Hg, Cd, Cr, As), X-ray, noise, etc. Premarital medical examination was used for couples to get married, in order to prevent diseases that might affect the health of offsprings and promote reproductive health, including the testing of serious hereditary diseases, infectious diseases, and psychiatric disorders. Chronic disease was defined as mothers or fathers had suffered from chronic diseases in 6 months before conception, such as heart disease, kidney disease, liver disease, hypertension, diabetes, anemia, etc. Housing renovation was defined as the house lived by mother had been renovated not more than 6 months. Strong tea drinking was defined as more than 200 ml per day on average. Pickled/smoked food intake, vegetable and fruit intake, fish/shrimp/meat/egg intake, and milk/soymilk intake were classified into three scales (times/week): ≤ 2, 3–5, >5, and the exposure time was defined as the first trimester of pregnancy. Smoking referred to active smoking in the study, and the exposure levels were classified into five scales (cigarettes/day): 0, 1–10, 11–20, 21–40, >40. Alcohol drinking was defined as drinking any liquor, including beer, wine and white spirit in the first trimester of pregnancy, the exposure levels were classified into three scales (times/week): 0, 1–2, ≥3.

*Characteristics and conditions of the fathers* In the present study, there were six variables related to the fathers, including age, occupational hazard exposure, chronic disease, smoking, alcohol drinking, and strong tea drinking. The definitions of paternal variables were the same as the maternal variables, and the exposure time was defined as 6 months before their wives’ conceptions.

### Quality control

We modified the questionnaire based on the pilot study. Before the formal survey, unified and strict training was provided to all of the investigators. The subjects were strictly selected according to the inclusion criteria and the diagnosis criteria. Five percent of all of the completed questionnaires were reviewed randomly, and the questionnaires with missing data >10 % and/or errors in logic >10 % were excluded from the study. To ensure the quality of the data entry, dual input was used, and logic checks were performed on the input data.

### Statistical analysis

A large number of variables (28 variables) were investigated in this study. We used univariate logistic regression to identify the NSCL/P-associated significant risk factors and then used Fisher discriminant analysis to establish a simple and useful prediction model based on the significant predictors. Univariate analysis could not control the confounding effect of other variables, or avoid the collinearity of some variables. Thus, in the Fisher discriminant analysis, we used a stepwise method to determine the final prediction, which could control the confounding effect and overcome the collinearity between variables.

Fisher discriminant was to find a linear combination for categorical groups, as the discriminant scores (Z) were calculated to maximize the between-group variance and minimize the within-group variation. The linear combination was known as a Fisher discriminant function as follows:

$$ Z={C}_1{X}_1+{C}_2{X}_2+{C}_3{X}_3+\cdots +{C}_m{X}_m $$

where *Z*: discriminant scores between two groups; *X*_1_, *X*_2_, *X*_3_, ⋯, *X*_*m*_: discriminant variables; *C*_1_, *C*_2_, *C*_3_, ⋯, *C*_*m*_ : discriminant coefficients for each discriminant variable. The discriminant variables could be selected via two methods: ‘enter variables together’ and ‘enter variables stepwise’. The stepwise method selected the discriminant variables on basis of Wilks’ lambda statistic, and in general, the F value was set at F _Entry_ = 3.84 and F _Removal_ = 2.71. The discriminant function established by stepwise discriminant was simpler and more effective. Assuming that the mean discriminant score of the controls was ${\overline{\mathrm{Z}}}_{\mathrm{A}}$, ${\overline{\mathrm{Z}}}_{\mathrm{B}}$ for the cases and $\overline{Z}$ for the total, then $\overline{Z}=\frac{{\overline{\mathrm{Z}}}_{\mathrm{A}}+{\overline{\mathrm{Z}}}_B}{2}$. According to the discriminant function, we calculate the discriminant score of *Z*_*i*_ for each subject; if *Z*_*i*_>$\overline{Z}$, the subject is considered highly likely to be a case, and if *Z*_*i*_≤$\overline{Z}$, the subject is regarded as a control.

Using Epidata 3.1 software (Jens M. Lauritsen, Michael Bruus and Mark Myatt, Odense, Denmark), we constructed a database and then entered the data. The data that were obtained were analyzed using SPSS 18.0 software (IBM, Chicago, IL, USA). The results were considered to be significant at *P* <0.05.

## Results

### Sociodemographic characteristics of the subjects

A total of 363 subjects who were admitted between July 2012 and June 2013 were surveyed (122 cases and 241 controls), and 24 subjects (9 cases and 15 controls) were excluded from the study because they refused to participate in the study, or the data collected was incomplete. Finally, 339 questionnaires were included in the study (93.4 % valid response rate), comprising 113 cases (92.6 % valid response rate, 34 cleft lip and 79 cleft lip with cleft palate) and 226 controls (93.8 % valid response rate). Table 1 shows the distributions of the sociodemographic characteristics of the two groups. Except for the maternal education level, no statistically significant differences were observed in the maternal age and occupation. The cases and controls were comparable, with good proportionality.

**Table 1:** Sociodemographic characteristics of the cases and controls

Characteristics	Controls (*n* = 226)	Cases (*n* = 113)	*χ* ^2^	*P*	OR^a^	95 % CI^b^ (OR)	
Maternal age (years), *n* (%)			0.983	0.805			
20–24	74 (32.7)	43 (38.1)			1		
25–29	96 (42.5)	45 (39.8)			0.807	0.481	1.352
30–34	51 (22.6)	23 (20.4)			0.776	0.418	1.442
≥ 35	5 (2.2)	2 (1.8)			0.688	0.128	3.702
Maternal education level, *n* (%)			6.460	0.033*			
Primary school and below	10 (4.4)	13 (11.5)			1.650	0.760	3.582
Middle school	160 (70.8)	79 (69.9)			4.673	1.476	14.795
College and above	56 (24.8)	21 (18.6)			1		
Maternal occupation, *n* (%)			6.708	0.243			
Farmer	88 (38.9)	39 (34.5)			1.723	0.756	3.928
Migrant worker	18 (8.0)	15 (13.3)			3.241	1.189	8.836
Business/company staff	35 (15.5)	9 (8.0)			1		
Worker	16 (7.1)	9 (8.0)			2.187	0.730	6.552
Staff in administrative institutions	22 (9.7)	13 (11.5)			2.298	0.843	6.267
House wife or else	47 (20.8)	28 (24.8)			2.317	0.971	5.526

^a^OR denotes odds ratio; ^b^CI denotes confidence interval; *P < 0.05

### Screening of the predictors

Using univariate logistic regression analysis, 28 variables were analyzed in sequence, including maternal and paternal variables relevant to NSCL/P.

Based on the univariate logistic regression analysis, the following 13 variables were significantly associated with NSCL/P (Table 2): low maternal education level, low family income, a premarital medical examination, a upper respiratory tract infection in the first trimester of pregnancy, complications of pregnancy, contraceptive intake before pregnancy, maternal occupational hazards exposure, housing renovation, fish/shrimp/meat/eggs intake, milk/soymilk intake in the first trimester of pregnancy, paternal occupational hazards exposure, paternal strong tea drinking, and the family histories of the parents. Among them, the premarital medical examination, fish/shrimp/meat/eggs intake and milk/soymilk intake in the first trimester of pregnancy were protective factors. The other 15 variables that were analyzed by the univariate logistic regression revealed no statistical significance, including maternal smoking. Rates of maternal smoking in the first trimester of pregnancy among cases and controls were 2.7 % (3/113) and 0.9 % (2/226), respectively. These five mothers smoked ‘1–10 cigarettes/day’.

**Table 2:** Results of univariate logistic regression analysis on influencing factors of NSCL/P

Screened factors	Controls (*n* = 226)	Cases (*n* = 113)	*b* ^c^	*P*	OR^a^	95 % CI^b^ (OR)	
Education level, *n* (%)				0.031*			
College and above	56 (24.8)	21 (18.6)			1		
Middle school	160 (70.8)	79 (69.9)	0.500	0.206	1.650	0.760	3.582
Primary school and below	10 (4.4)	13 (11.5)	1.542	0.009*	4.673	1.476	14.795
Family income (Yuan/year/person), *n* (%)				0.003*			
> 15,000	22 (9.7)	6 (5.3)			1		
10,001–15,000	10 (4.4)	1 (0.9)	−1.003	0.381	0.367	0.039	3.462
5001–10,000	107 (47.3)	39 (34.5)	0.290	0.056	1.336	0.504	3.541
≤ 5000	87 (38.5)	67 (59.3)	1.038	0.034*	2.824	1.084	7.355
Premarital medical examination, *n* (%)	56 (24.8)	13 (11.5)	−0.930	0.005*	0.395	0.206	0.757
Upper respiratory tract infection, *n* (%)	75 (33.2)	55 (48.7)	0.647	0.006*	1.909	1.204	3.028
Complications of pregnancy, *n* (%)	6 (2.7)	9 (8.0)	1.155	0.033*	3.173	1.100	9.149
Contraceptive intake before pregnancy, *n* (%)	2 (0.9)	6 (5.3)	1.837	0.026*	6.280	1.247	31.634
Maternal occupational hazards exposure, *n* (%)	5 (2.2)	16 (14.2)	1.987	0.000*	7.291	2.597	20.466
Housing renovation, *n* (%)	11 (4.9)	21 (18.6)	1.495	0.000*	4.461	2.067	9.629
Fish/shrimp/meat/eggs intake, *n* (%)				0.033*			
≤ 2 times/week	16 (7.1)	15 (13.3)			1		
3~5 times/week	79 (35.0)	48 (42.5)	−0.434	0.282	0.648	0.294	1.429
> 5 times/week	131 (58.0)	50 (44.2)	−0.899	0.023*	0.407	0.187	0.885
Milk/soymilk intake, *n* (%)				0.000*			
≤ 2 times/week	26 (11.5)	35 (31.0)			1		
3~5 times/week	82 (36.3)	36 (31.9)	−1.120	0.001*	0.326	0.172	0.619
> 5 times/week	118 (52.2)	42 (37.2)	−1.330	0.000*	0.143	0.143	0.490
Paternal occupational hazards exposure, *n* (%)	3 (1.3)	14 (12.4)	2.352	0.000*	10.512	2.954	37.402
Paternal strong tea drinking, *n* (%)	20 (8.8)	20 (17.7)	0.795	0.019*	2.215	1.138	4.313
Family history of NSCL/P, *n* (%)	2 (0.9)	13 (11.5)	6.678	0.000*	14.560	3.225	65.728

^a^OR denotes odds ratio; ^b^CI denotes confidence interval; ^c^b denotes partial regression coefficient; *P < 0.05

### Establishment of the prediction model

Using the results of the univariate logistic regression analysis, a risk prediction model of NSCL/P was constructed by a stepwise Fisher discriminant analysis (F _Entry_ = 3.84, F _Removal_ = 2.71) based on the screened 13 variables that were statistically significant. The stepwise discriminant analysis showed that Wilks’ lambda, as a test of the discriminant function, was significant (lambda = 0.772, chi-square = 86.044, df = 8, *P* < 0.001), and 8 variables were selected, as follows: family income (*X*_1_), maternal occupation hazards exposure (*X*_2_), premarital medical examination (*X*_3_), housing renovation (*X*_4_), milk/soymilk intake in the first trimester of pregnancy (*X*_5_), paternal occupational hazards exposure (*X*_6_), paternal strong tea drinking (*X*_7_), and the family history of NSCL/P (*X*_8_). The final standardized discriminant function was calculated according to the following Equation:

$$ Z=-0.287{X}_1+0.283{X}_2-0.255{X}_3+0.464{X}_4-0.338{X}_5+0.309{X}_6+0.236{X}_7+0.422{X}_8 $$

In the discriminant analysis, ${\overline{\mathrm{Z}}}_{\mathrm{A}}$ = −0.383, ${\overline{\mathrm{Z}}}_{\mathrm{B}}$ =0.766, and $\overline{\mathrm{Z}}$ = (0.766–0.383)/2 = 0.192. Then, we calculated the discriminant function value of *Z*_*i*_ for each subject; if *Z*_*i*_>0.192, the subject was considered highly likely to be a case of NSCL/P, and if *Z*_*i*_≤0.192, the subject was regarded as normal.

### Prediction of the discriminant analysis predictive effect

#### Accuracy of prediction

The prediction of the accuracy of the prediction model was performed by self-verification. Table 3 shows the results of the classification of the self-verification. 83.8 % of the subjects were correctly classified as either a NSCL/P case or a control, the rates of correct prediction were 74.3 % for the NSCL/P cases (sensitivity) and 88.5 % for the controls (specificity), and the positive and negative predictive values were 76.4 and 87.3 %, respectively.

**Table 3:** Classification results of self-verification

Actual classification	Predicted group membership		Total
	Control (%)	Case (%)	
Control	200 (88.5)	26 (11.5)	226
Case	29 (25.7)	84 (74.3)	113
Total	229	110	339

1. There were 200 controls and 84 cases (83.8 %) correctly classified (*n* = 339)

2. The positive and negative predictive value was 76.4 and 87.3 %, respectively

#### ROC curve analysis of the discriminant analysis prediction

An important measure of the accuracy of the prediction model is the receiver operating characteristic (ROC) curve. The area under the ROC curve (AUC) is typically between 0.5 and 1.0. When the AUC is between 0.5 and 0.7, the diagnostic value of the test is low; when it is between 0.7 and 0.9, it has a medium diagnostic value; and when it is more than 0.9, it has high diagnostic value.

**Fig. 1:**
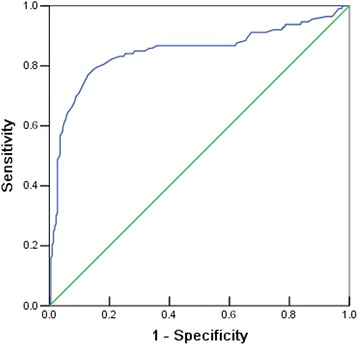
Receiver operating characteristic (ROC) curve of the discriminant analysis prediction model

The AUC of the discriminant analysis prediction model is shown in Fig. 1. The AUC demonstrated statistical significance (AUC = 0.846, SE = 0.027, *P* < 0.001, 95 % CI: 0.794~0.898). The diagnostic value of the model was medium.

## Discussion

NSCL/P is a common congenital anomaly, which seriously affects children’s health. The etiology of NSCL/P is complex and largely unknown. Recently, most studies have focused on the identification of risk factors of NSCL/P, while an effective risk prediction tool for NSCL/P is lacking. In the present study, the prediction model established by discriminant analysis was successful in classifying 83.8 % of participants, with an AUC of 0.846. The prediction model can be used as a risk prediction tool for NSCL/P, as it aims to identify the high-risk population of NSCL/P in the first trimester of pregnancy and to provide important information for a further clinical ultrasound in the second or third trimesters of pregnancy. The pregnant women with a high predictive risk were identified as the population at a high risk of NSCL/P and listed as a focus group for clinical prenatal ultrasound diagnosis. In addition, the prediction model also can be applied by doctors into pre-conception counseling and education for women of childbearing age. If women of childbearing age discover that they are at a high risk by this prediction, they may be able to control some important risk factors to reduce the risk of NSCL/P during pregnancy. To the best of our knowledge, there was no available information on predicting the occurrence of NSCL/P. Accordingly, this is the first study using a discriminant analysis to predict the risk of NSCL/P in pregnant women.

In the present study, 13 factors screened by univariate logistic analysis were related to NSCL/P, but only 8 factors used as predictor entered the discriminant function. Consistent with previous studies, a low family income [14], not attending premarital medical examinations [35], family history [14–16], maternal occupational hazards exposure [21, 22] and paternal occupational hazards exposure [23] selected as predictors were significantly associated with NSCL/P. According to Krapels et al. who examined maternal nutritional factors related to orofacial cleft in Netherlands, increasing intake of vegetable protein can decrease the risk of orofacial cleft [36]. Shaw et al. found that decreased NSCL/P risk was associated with increased intake of total protein [25]. In China, a case–control study conducted in Hubei Province showed that maternal diet of eggs or milk in first trimmest of pregnancy was significantly associated with a decreased risk of NSCL/P [23]. Similar result was found in our study, showing that milk/soymilk intake in the first trimester of pregnancy was significantly related to NSCL/P. In addition, we also found that housing renovation and paternal strong tea drinking were significantly associated with NSCL/P. Consistent with our findings, a previous observational study found that paternal strong tea drinking was significantly associated with an increased risk of birth defects in offspring [37]. The reason for paternal strong tea drinking increasing the risk of NSCL/P might be attributed to caffeine, which was a plant alkaloid in teas. Evidences from both animal experiments and human studies [38–40] demonstrated that the intake of caffeine and caffeinated beverages among males could impair reproductive organs, sperm characteristics, and sperm quality, affect fertility and fetal health, and even cause birth defects. Eight predictors selected by a discriminant analysis were with good representativeness and availability.

In this study, the NSCL/P risk prediction model had good specificity, while the sensitivity was not satisfactory. The sensitivities (the rates of correct prediction for the NSCL/P cases) and the specificities (the rates of correct prediction for the controls) were 74.3 and 88.5 %, respectively. There were two reasons for the low sensitivity. First, the 8 predictors selected by the discriminant analysis except for the family history were common risk factors of congenital anomalies but were not specific indicators for NSCL/P. Second, due to the small sample size and the low exposure rates of some of the investigated factors, some common important risk factors were not included in the prediction model, such as maternal age, folic acid intake, history of infection during pregnancy, mothers’ abnormal reproductive history, medication use during pregnancy, maternal stressful events during pregnancy, tobacco, and alcohol. Many of the published papers show conflicting results on the relationship between maternal age and NSCL/P [10, 12, 41]. The effect of folic acid on NSCL/P has generated debate in previous studies [28, 41, 42]. The results from the present study showed that maternal age and folic acid intake were not significantly related to the occurrence of NSCL/P, which is consistent with the findings of Golalipour’s study [41] conducted in Iran and Bille’s study [28] conducted in Denmark.

The present study has specific limitations. First, we used case–control data to select the predictors, and this inevitably led to recall bias in the data. Second, because of the limitations of the sample size, a self-verification was adopted to evaluate the discriminant predictive effect of NSCL/P, which tended to exaggerate the discriminant effect. Further studies are needed to confirm the validity and reliability of the NSCL/P prediction model in the larger population. Third, the 95 % confidence intervals (CI) of odds ratios (OR) for some of the screened factors (e.g. maternal occupational hazards exposure, paternal occupational hazards exposure, and family history of NSCL/P) were wide due to the small sample size. The corresponding ORs were significant, but with limited precision and reliability. Finally, due to the low exposure rates of some of the investigated factors, certain important risk factors of NSCL/P failed to enter the prediction model, resulting in its low sensitivity. We will need to conduct additional research to identify the specific predictors of NSCL/P to improve the sensitivity and specificity of the model and attempt to construct the prediction model by other statistical methods, such as artificial neural networks, decision trees or logistic regression, to modify and improve the prediction model.

## Conclusions

The discriminant prediction model, which is based on family income, maternal occupational hazards exposure, premarital medical examination, housing renovation, drinking milk/soymilk in the first trimester of pregnancy, paternal occupational hazards exposure, paternal drinking of strong tea, and family history of NSCL/P, is useful for predicting the risk of NSCL/P. Further research is needed to improve the model, and confirm the validity and reliability of the model.

## Abbreviations

AUC: Area under the ROC curve
CI: Confidence interval
NSCL/P: Non-syndromic cleft lip with or without cleft palate
OR: Odds ratio
ROC: Receiver operating characteristic
